# School-Related Factors in the Implementation of a Positive Youth Development Project in Hong Kong

**DOI:** 10.1100/tsw.2008.121

**Published:** 2008-10-10

**Authors:** Daniel T. L. Shek, Yammy L. Y. Chak, Candace W. Y. Chan

**Affiliations:** ^1^ Quality of Life Centre, Hong Kong Institute of Asia-Pacific Studies, The Chinese University of Hong Kong, Hong Kong; ^2^ Department of Sociology, East China Normal University, Shanghai, China; ^3^ Kiang Wu Nursing College of Macau, Macau

**Keywords:** positive youth development, Project P.A.T.H.S., project implementation, process variables, Chinese adolescents

## Abstract

Individual and focus group interviews were conducted to identify school-related factors that influence the process and quality of implementation of the Tier 1 Program of the Project P.A.T.H.S. in Hong Kong. Results of this case study approach showed that the program implementation quality was generally high. Factors that facilitate the implementation of the program were identified, including administrative support from the school and social work agency, presence of dedicated teachers, positive perceptions of the program among teachers, the teachers' self-disclosure, effective continuous assessment, and excellent co-teaching mode. Difficulties encountered by the school in the process of implementation were also observed. Based on the present findings, school-related process variables that facilitate or impede the implementation of positive youth development programs in the Chinese context are discussed.

### INTRODUCTION

The Project P.A.T.H.S. (**P**ositive **A**dolescent **T**raining through **H**olistic **S**ocial Programmes) was designed to promote positive development in Chinese adolescents in Hong Kong. Although the effectiveness of the project has been documented in previous studies[1,2,3,4], there is a need to understand more about the process variables that influence the quality of program implementation. Actually, few studies have examined factors that contribute or impede the implementation of positive youth development programs in both international and Chinese contexts.

Researchers have proposed many attributes of effective prevention programs[5,6,7,8,9]. These include comprehensiveness, good theoretical foundation, use of diverse teaching methods, sufficient program intensity, positive relationship orientation, sociocultural relevance, developmental appropriateness, presence of well-trained staff, and outcome evaluation. In contrast, less work has focused on the effect of school-related characteristics in program implementation. As such, school-related factors that facilitate or impede the implementation of positive youth development programs were examined in this study.

Studies showed that support by school principals contributed to successful program implementation[10,11,12]; the availability of a key person to coordinate and conduct the program was also important[13]. Principals and/or coordinators who were encouraging and able to provide clear guidance and information to teachers were instrumental to the program's success[6,13]. Moreover, research findings showed that teachers who understood and identified with the program's philosophy favored effective program implementation[5,6,8]. Other teacher variables, including teacher self-efficacy beliefs, professional burnout, acceptance of the program, similarity of the program with the teachers' belief in the students' behavior, and the perceived effectiveness of the program influenced the teachers' motivation to implement the program, and hence the quality of program implementation[12].

Besides "people" factors, school administrative and policy issues, such as manpower deployment, were related to the implementation of the school-based program[5,7]. In particular, effective program implementation occurred when the program was incorporated into the school's curriculum and implemented with high program fidelity[6,9]. The incorporation of a new program into an existing school curriculum requires changes in the curriculum, administrative arrangement (e.g., teaching timetable), and the teachers' beliefs and attitudes[14,15]. One thorny issue is resistance from teachers regarding the implementation of the program. While teachers might verbally agree that positive youth development programs are important, they usually lack the motivation to implement them. To examine the underlying reasons why teachers did not consistently act out their expressed beliefs, Keys[14] showed that lack of ongoing professional support, resources, and time contributed to the observed phenomenon. In the first place, the lack of ongoing professional support, such as professional development and administrative support, hindered the teachers' willingness to bring their expressed beliefs into teaching practice. In addition, lack of resources, such as new equipment and teaching materials, would impede the teachers' motivation to embrace the change. Finally, time is another factor that is used by the teachers as an excuse for not planning or fully implementing the program. Teachers usually argued that they had to spend too much preparation time in planning and implementing the new program.

Implementation format is another factor that influences the quality of program implementation. Research findings showed that students and parents had positive perceptions about coteaching among teachers, and they perceived cotaught classes as more interesting and favorable[16]. Unfortunately, despite the significance of coteaching in educational research, few studies have examined the influence of coteaching on the implementation of school-based programs. Research findings have shown that classroom discipline could be better managed and program content could be delivered more effectively under coteaching[17]. Studies also showed the beneficial effect of coteaching in the students' achievement[16,17,18,19]. However, Sawyer and Rimm-Kaufman[20] reported that teacher collaboration was related to several factors, including the teachers' perceptions of the school environment, perceptions of shared educational goals and values among teachers, and the perception of barriers to collaboration. Lack of time and lack of administrative priority were also considered to be barriers to teacher collaboration, although the significance was controversial[20]. Moreover, teachers were more willing to collaborate with other teachers in their same grade level.

Finally, effective delivery via effective teaching strategies is also instrumental to successful program implementation. The use of varied teaching strategies (such as audio-visual materials, worksheets, and role play), interactive teaching strategies[8], and appropriate translation of program concepts in the schools lives of the students is important[10]. Moreover, the teachers' self-disclosure was a significant element that elicited the students' participation in class[21]. Goldstein and Benassi[21] pointed out that the teachers' self-disclosure did not only act as an example of the course concepts, but also encouraged student involvement by creating an open atmosphere in class. There are studies that document the therapeutic value of worker's self-disclosure in counseling contexts[22,23,24]. Obviously, such benefits may also be generalizable to educational contexts provided that the professional guidelines on self-disclosure were followed[25].

Utilizing a case study approach, the present study attempted to identify school process variables that might influence the quality of implementation of a positive youth development program in Hong Kong. Based on the study, school-related factors that facilitate or impede the implementation of the program were identified. Besides enriching theoretical models on process variables, the related findings also have implications for future school-based program implementation.

### METHODS

#### Participants

For the academic year 2006/07, 207 schools joined the Project P.A.T.H.S. in the Full Implementation Phase (Secondary 1 level). Among all these schools, seven schools with positive and effective implementation were identified and invited to participate in this case study after completion of the Tier 1 Program. The implementation characteristics of one of these schools are documented in this paper.

#### Procedures

A qualitative research design was adopted in this study, involving one individual interview with the school contact person (i.e., school coordinator for the Project P.A.T.H.S.) and a focus group interview with four teachers (two males and two females) who implemented the Tier 1 Program. Consent from the school principal and the informants was sought prior to the study. Before conducting the interview, the school coordinator completed a questionnaire on the background information of the school.

The second author and a trained colleague with a Master degree in Social Work conducted the interviews. All interviews were conducted in Cantonese with semi-structured open-ended questions. There were four sections in the semi-structured interview schedules: (1) informants' perceptions of the school administrative arrangement, such as preparation, support within the school and from the social work agency, and program evaluation; (2) informants' perceptions of the program implementation process; (3) informants' perceptions of the program effectiveness, program success; and (4) respondents' overall impression. Follow-up questions were added to elicit more information and thought if new insights emerged.

#### Data Analyses and Case Writing

The individual interview and focus group interviews were tape recorded and subsequently transcribed. A general qualitative analyses technique[26] was used to identify major themes. There were three phases of data analysis: data reduction, data display, and conclusion drawing with verification. An inductive analysis and content analysis were conducted to identify categories and themes emerging from all interviews. To ensure reliability in the interpretation process, the preliminary analyses conducted by the second author were further checked by the first author to ensure the accuracy and trustworthiness of the qualitative data. In addition, the authors compiled reflections on personal biases, expectations, and assumptions that might influence the interpretation of data.

### RESULTS

The characteristics of the school are shown in Table 1. A Life Education team consisting of four teachers and a teaching assistant (with a Bachelor degree) conducted and implemented the Tier 1 Program in the school. The team had been assigned to coteach in the lessons of Life Education since 2003. Before joining

**TABLE 1**
**Summary of the Characteristics of the School**

| | |
|---|---|
| **I. Basic Information of the Program** | |
| Program details | 190 students (five classes); 20-h full program with 40 30-min units |
| Class teacher system | Double class teachers system |
| Program arrangement | Life Education (100%, about once every 2 weeks); |
| Introduction session and conclusion session | Yes |
| Student assessment on P.A.T.H.S. Program | Yes (homework, group project, and examination in Life Education) |
| No. of instructors who conducted Tier 1 Program (and teachers who joined training) | Four teachers and one teaching assistant (three teachers) |
| **II. Basic Information of Tier 1 Program Implementation** | |
| No. of preparation meetings and content | One formal preparation meeting was scheduled before the beginning of the first semester, where there was discussion on the implementation aims, course content, arrangement, and related matters. |
| No. of instructors who conducted Tier 1 Program in each class | Two teachers |
| Person responsible for preparing the teaching material and the way of preparation | The teaching assistant prepared all teaching materials and worksheets for the teachers. |
| The format and frequency of lesson preparation among instructors/Reasons for not preparing the lessons together | No formal and regular lesson preparation meeting. The school coordinator helped to prepare all teaching plans and reserved all the audio-visual aids. The teachers discussed before each lesson informally as they reflected that there was no time to have meetings. |
| The format and frequency of experience sharing after class among instructors/Reasons for not sharing | No formal and regular meeting. The teachers discussed together and shared their experiences after each lesson informally. |
| No. of evaluation meetings and content | One final evaluation meeting, where there were reports on the issues related to preparation and implementation, the difficulties encountered, and follow-up. |
| Cooperation between the school and social work agency | The school was responsible for the Tier 1 Program, while the social work agency was responsible for the Tier 2 program only. |
| The role of teacher in Tier 1 Program implementation | i. Teaching the program; ii. Monitoring classroom discipline; iii. Preparing teaching materials; iv. Coordinating the program-related matters |
| The role of social worker in Tier 1 Program implementation | i. Coordinating the program-related matters; ii. Following-up |

the Project P.A.T.H.S., the team put in a lot of effort to develop their own school-based curriculum for the subject. After adopting the program, they combined the program of the Project P.A.T.H.S. with their existing curriculum (the team selected and kept some good and attractive topics) and tailor made a “new and comprehensive curriculum” to perfect the subject. Moreover, students were required to take examinations on Life Education content covered at the end of each term.

Due to heavy workloads and busy schedules, the team only arranged one formal preparatory meeting before the start of the first term, where there was discussion on the aims, course content, arrangement, and related matters, in order to become familiar with the curriculum content. During the implementation, they managed to meet each other informally either before or after running the activities in classes, or at lunch, to discuss the units, roles, and responsibilities, as well as the difficulties encountered and follow-up.

In the implementation process, a school coordinator was responsible for coordinating the administrative arrangement, helping to prepare teaching plans, and reserving all the audio-visual aids. In addition, a teaching assistant was responsible for preparing all teaching materials for the teachers. In the Tier 1 Program, the role of teachers was (1) teaching the program, (2) monitoring classroom discipline, (3) preparing teaching materials, and (4) coordinating the program-related matters. On the other hand, the social worker was mainly responsible for coordinating the program-related matters and following-up with students.

### School Administrative Arrangements and Related Issues

The results of the school administrative arrangements and related issues are presented in Table 2. Several observations as far as school support for the program can be highlighted. First, high support from the school contact person was very important. Because the school coordinator supported the project and its rationales, she initiated the implementation of the program in her school. Second, the support from the teachers was also significant because it had a positive influence on the participation and effective implementation. Third, the teachers considered the program to be good and practical for the students, and this perception was also essential when facilitating the program implementation.

Concerning the perceived program arrangements, all teachers considered this to be an effective and good practice, as the program was arranged to fit into the existing school's lessons with similar content, which did fit them, as well as fit their students best. In other instances, the teachers expressed that they could make use of some previously prepared school-based materials (with high quality) while implementing the program in order to perfect the subject of Life Education. Furthermore, one teacher pointed out that as they were the one who could master the content of the subject of Life Education and the Project P.A.T.H.S., this was another advantage to incorporating the program into the formal curriculum, as they could keep the teaching quality and lessen the variation. The team members also perceived that this kind of implementation was the same as other school subjects and, thus, would not affect the school system and others.

The findings revealed that the school had effective manpower deployment and good teamwork. First, the school coordinator was the chief member to oversee the program implementation in the school and was responsible for communication with the Life Education teachers. Second, there was a team of Life Education teachers to carry out teaching. Third, there was a teaching assistant who had to support the Life Education teachers in order to create more free time for them. The consolidated interpretations can be seen in Table 2.

The next issue is related to equipment and facilities. From the interview, the teachers raised the concerns that the equipment and facilities for program implementation were not sufficient. The school coordinator did encounter difficulties in arranging the audio-visual aids. This problem is reflected in the narratives of Teacher C:

> *"We've got computers in every classroom, although it's slow. Concerning the projectors, we can use them after making reservation, but, it bothers our workmen … Actually, only two stories of the classrooms have installed projectors in this school."*

Finally, support within the school and from the social work agency was instrumental to successful program implementation (see Table 2). As revealed in the interviews, good support within the school was found. The team perceived that they had support from the previous and present principals, as both principals had consistently provided sufficient manpower for coteaching in this subject. Regarding other teachers in the school, although other subjects' teachers did not fully understand the program, the team took the initiative to share experiences and information with the others to let them understand the project. For this reason, a supportive and positive culture was created among different teachers. In addition, the support from the social work agency was also adequate. This can be seen in the narratives of Teacher A:

**TABLE 2**
**Consolidated Interpretations on the Administrative Arrangements and Related Issues in the School**

| Aspect | Integrated Findings and Interpretations |
|---|---|
| (a) Support for the program and its rationales | • High support from the school contact person, which facilitated program implementation.<br>• The team of Life Education teachers supported the project, especially incorporation of the program into the formal curriculum.<br>• The teachers considered the program to be good and practical for the students. |
| (b) Perceived program arrangement, and its advantages and disadvantages | • The program was incorporated into the existing school system.<br>• The program was arranged to fit into the existing school lessons with similar content.<br>• Advantages of incorporation: (a) Used existing time slots for teaching; (b) the program topics could merge with the existing and the previous school-based curriculum; (c) could ensure the teaching quality of the subject; (d) had smooth implementation and good arrangement that did not disturb the school system and affect others.<br>• Disadvantages of incorporation: (a) previous tailor-made teaching materials made by the team could not be totally used; (b) as the subject teachers, teachers felt it difficult to be familiar with all their students; (c) the content was too broad and sometimes vague. |
| (c) Manpower deployment | • The manpower distribution in Tier 1 Program was:<br>1. One chief coordinator had to oversee the program implementation and communicate with the Life Education teachers.<br>2. Four teachers had to teach five S1 classes and five S2 classes.<br>3. One teaching assistant (with a Bachelor degree) was responsible for all preparation and coordination. |
| (d) Facilities for implementation | • No sufficient equipment and facilities facilitated the implementation. |
| (e) Support within school and from social work agency | • Attributes of the school principal: supportive and trusted her colleagues.<br>• Attributes of the school contact person: supportive and allowed collaborative decision making.<br>• Qualities of the teachers: cooperative, supportive, and had initiative to share experiences and information.<br>• The school had a good and clear collaborative relationship with the social work agency.<br>• Communication among different parties was good.<br>• Other subjects' teachers did not fully understand the project. |
| (f) Impression on the overall administration | • The school coordinator reflected that there were not many difficulties encountered. |

*"Actually, the relationship between the NGO and our school are very good; we both have a clear consensus. I understand that if we ask for their assistance for the Tier 1 Program, which means we should cut the resources of the Tier 2 Program. Thus, we both prefer to work on our specialty."*

### Implementation Characteristics in the School

The integrated findings and interpretations related to the implementation characteristics in the school can be seen in Table 3. As regards the students' responses, the school coordinator indicated that all students were told by the team in the lessons that the school had incorporated the program into the subject of Life Education. A teacher highlighted that as the most important characteristic of the program was the abundant activities, active participation from students was received. The team believed that the students considered the program to be fun and they found the program more interesting than their regular subjects. The team indicated that the students liked the video clips, soundtracks, group activities, and role play; they were eager to take part. In addition, the team found that all students were interested in the teachers' personal stories. Nevertheless, the teachers found that some of the students were passive and not involved in the lessons. The teachers also pointed out that some students felt bored when attending intensive classes, especially discussing "life meaning and values".

**TABLE 3**
**Consolidated Interpretations on the Implementation Characteristics in the School**

| Aspect | Integrated Findings and Interpretations |
|---|---|
| (a) Teachers' and students' responses in the implementation process | • Teachers viewed the program as common practice and everyone was accustomed to it.<br>• Teachers preferred teaching the lessons by themselves, not by social workers.<br>• Teachers had quite positive comments on the program.<br>• Students liked different activities. Their interest and involvement varied, and was dependent on the topics.<br>• Some of the Form 2 students were passive.<br>• Students were interested in teachers' personal stories and sharing. |
| (b) Coteaching | • Teachers cooperated and trusted each other in coteaching.<br>• Teachers enjoyed the process of coteaching.<br>• Good and supportive team work was established.<br>• Teachers with counseling training were a better choice to run the program. |
| (c) Teaching techniques | • Technique 1: Teachers' personal sharing.<br>• Technique 2: Have a good consolidation before the end of each lesson.<br>• Technique 3: Serious in marking student worksheets and giving feedback. |
| (d) Handling students' worksheets | • Checking students' assignments was beneficial.<br>• Student folders were used. |
| (e) Student assessment in the Tier 1 Program | • Continuous assessment was exercised with a good effect.<br>• Life Education has written examination. |
| (f) Difficulties encountered and solutions | • The school contact person did not perceive any big difficulties, but felt strained due to the arrangement of facilities.<br>• Difficulties expressed by teachers:<br>1. Difficulties in monitoring highly active students<br>2. Insufficient time to run activities<br>3. Overlap of the topics<br>4. Strange linkage between some themes and topics<br>5. Difficulties in preparing examination papers<br>6. Problems in preparing the audio-visual aids |
| (g) Advice for next year's implementation | • Periodically update the teaching materials, and search for more relevant and latest information to meet students' needs.<br>• Have a clear index inside both teaching manuals. |

Concerning the teachers' responses, the teachers viewed that the program had become "common practice" in the school and everyone had become accustomed to it. For this reason, it helped the team to feel more secure when delivering the program. In addition, the team had quite positive comments on the program and they preferred to teach the lessons by themselves, not by the social workers (because of different professionalism), in order to have effective teaching and time management. The perceptions of teachers on interdisciplinary collaboration are shown in the narratives of different teachers:

> *Teacher A: "As to administrative issues, to be honest, there are a few things I need to know before I will allow my students to be taught by a particular person: whether we can work together in meetings, what kind of social workers they are sending etc. I cannot have peace of mind until I get such information. We have very tight time frame, about 40 minutes, during which we have to try to work together. The students may take advantage of the social workers or test his/her abilities. As a result, there is not enough time for teaching."*

> *Teacher C: "I don't think there's a need to change, as sometimes it is difficult for social workers to manage the classroom discipline… I believe that we (as teachers) have the professional skills to manage the classroom discipline, and I believe that everything will be smooth."*

> *Teacher D: "I think the level of trust is very important, I don't mean that the social worker is not reliable, but after a period of time, we have developed a good relationship with the students, and so we are willing to share with them about our personal and growth stories … but I think it's hard for social workers to catch the attention of the students to listen their sharing."*

In addition, the team mentioned that teachers with counseling training were a better choice to run the program.

> *Teacher C: "we are the counseling teachers. We are the one with counseling experiences; I think we are the better choice of persons. It is because different teachers have different characters and abilities, and not everyone can get the job done."*

Regarding teaching strategies that were beneficial to the program implementation, the informants suggested several means. These included: (1) to have a good consolidation for each lesson; (2) to be serious in marking students' worksheets and giving feedback, and (3) to ask the students to hand in completed worksheets and remind them to keep all their worksheets in the folders, which in turn let the students become serious and motivated.

The teachers also revealed that coteaching was desirable and they enjoyed the process of coteaching. Good and supportive work relationships were established. Some characteristics of coteaching in this school were found. First, the teachers had fixed partners and took turns. Second, the teachers teamed up with one another in the class. It was common to see either teacher presenting to the class as a whole, while the other teacher would interject with elaborations. Third, the team had good teamwork, and the teachers appreciated the fellowship of their team. As mentioned by Teacher C:

> *"We have worked together before and there is a collaborative spirit among us, so that I will know what you mean after you utter your first sentence. I may even be able to follow up with another statement. These kinds of cooperation are really gratifying!"*

More importantly, the informants suggested that teachers could personalize their teaching through the use of humor, personal stories, and experiences in order to attract students. In particular, the informants found self-disclosure to be an effective and practical way to convey messages, as most of the students were curious to know what was happening to others, especially their teachers.

> *Teacher D: "However, probably, the students can remember teacher's own sharing the most. For example, there is a unit in Form 1 called "My Favorite" which is about what one treasures most in life. This unit can help to lessen the materialistic desire of the students. I told a story about the incident of Pak Sin Leng. I told them it was actually not a written story but a real story instead. Then I told the whole story… by the time of the Pak Sin Leng accident happened, I was still studying in school, my teacher told us if you would become a teacher, would you be willing to do something like this? Then I shared with my students about my thoughts… And then, I even told them that I tried to write a self recommendation letter to that school to apply a job there as I thought the students there may have changed to be better. I told them the Principal of that school did ignore me and they laughed. So I think they could then remember what message I have conveyed as they are all curious to know what's happening on others."*

Finally, according to the school coordinator, to enable better teaching quality, the teams of teachers were selected with suitable characteristics and a "mission" to carry out the lesson. In fact, the teams also expressed that the principal selected those colleagues with "heart" for this program".

As the Tier 1 Program was incorporated into the formal curriculum, the informants generally suggested that assessment was helpful. The school coordinator reported that continuous assessment was exercised with good effect, including written examinations (50% of the subject weighting), assignment and group projects, assessment of performance by their form teachers as well as subject teachers, and the students' self-assessment. For the written examination, the informant stressed that written examination focused on testing application, not memorization.

### Perceived Program Effectiveness, Program Success, and Overall Impression

As reflected from the evaluation questionnaires in the Experimental Implementation Phase, the school coordinator admitted that the program was beneficial to the students and their responses were positive. Since there were various school activities and many factors affecting the students' development and growth, the coordinator was unsure if the changes in all students were totally related to the program. Although she had no obvious answer on program effectiveness, she still recognized the program to be successful because she discovered that some of the students showed growth with improvement in the lessons.

On the other hand, the informants perceived that the program might be beneficial to the school atmosphere, as reflected in the narratives of two teachers:

> *Teacher A: "we found that the negative thought of students towards things around is lessened, and they know how to cherish everything. I believe there is improvement… and this is what the program has contributed."*

> *Teacher C: "The atmosphere of the whole school has improved, and the appearance of the violent behaviors were diminished."*

These findings show that different people had diverse definitions of success, which generated different answers to the questions of perceived program success. Generally speaking, even though the team considered that the program was not the only contributing factor to facilitate the students' change, the team had positive impressions of the program. They both perceived the program to be diverse, comprehensive, and useful to their students.

## DISCUSSION

The purpose of the present paper was to identify major school-related process variables that influenced the quality and success of program implementation. Based on this case study, several factors were identified.

### Sufficient School Administrative Support

This case reveals that incorporation of the Tier 1 Program into the curriculum is an ideal arrangement as all people, either teachers or students, would treat the program as a subject. In order to have effective instruction, the principals (previous and present principals) provided sufficient manpower for coteaching consistently. In addition, an organized manpower deployment, enough support for teachers, and the creation of a structural team for program implementation with specific roles and responsibilities were also the significant contributing factors. Murawski and Dieker[27] pointed out that administrative support for coteaching at the school is very important and significant. The present findings concur with their views. Gabriel[28] described those who build and maintain a successful team, and facilitate professional growth of teachers as "teacher leaders". Lieberman[29,30] suggested that teacher leaders can build trust and rapport, manage resources, coordinate work, demonstrate expertise, and share knowledge with others. The findings provided support that the coordinator and some of the teachers showed these qualities.

### Involvement of Dedicated Teachers

The present case suggests that dedicated teachers who have a passion for students are important to program success. Although the teachers faced a challenge in adopting a new program and putting away their well-prepared teaching materials, they were committed and enthusiastic to develop a new curriculum and change their mind set for "re-engineering" the Life Education curriculum. Attributes that could be highlighted from the interviews were an enthusiastic team, humanistic and experienced colleagues, responsible and self-motivated teachers, good collaborative relationships, and mutual support among the team. Although difficulties (e.g., extra workload in preparation, inadequate equipment in every classroom) were encountered in the implementation process, the team helped the colleagues to maintain their devotion and enthusiasm, so that they could then have strengths and peer support as motivators, and solve the difficulties effectively.

### Positive Perception of the Program

The findings showed that the teachers had positive perceptions towards the program goals and they regarded the program to be beneficial to student development. In this case, voluntary participation in the program tended to elicit less resistance and resentment. The teachers also supported the program and agreed with its rationales. This laid a good foundation and exerted a significant influence on effective program implementation.

### Genuine Teachers' Self-Disclosure

The team reflected that they were keen and prepared to have self-disclosure. As students generally desired the teachers' self-disclosure, this team of teachers was willing to share their personal views, positive or negative life experiences, and growth stories with their students during the lessons. The psychological literature suggests that interpersonal attraction is related to degree of communication as well as selfdisclosure; self-disclosure can also change the direction, definition, and intensity of relationships[31]. Tardy and Dindia emphasized that self-disclosure can facilitate relationship imitation, development, and hence relational maintenance[32]. There are also findings that suggest that self-disclosure of therapists can foster alliance, egalitarian relationships, role and skills modeling, client insight and learning, informed choice, and moral solidarity[24]. Consistent with the literature, it was observed that the teachers' self-disclosure contributed to classroom participation as well as student-teacher alliance, and even encouraged interaction among students. However, when workers utilize self-disclosure in positive youth development programs, they should be aware of the complexity involved. Hopmeyer[25] proposed 12 guidelines for using self-disclosure, including positive use, consistency with the teaching objectives, and utilization within the boundaries of privacy and personal comfort. Knox and Hill[33] also outlined several principles governing self-disclosure among helping professionals. Obviously, when using self-disclosure in class, teachers should clearly identify the boundaries and roles, and carefully determine the suitable moment for disclosure.

### Excellent Coteaching Relationship

The findings underscore the importance of deciding to use coteaching in the program, which made the program successful. Yet, the relationship between the coteachers is a key component that influences the success of the coteaching. Gately and Gately[34] emphasized that effective interpersonal communication was one of the important components of the coteaching relationship, which was reflected in the present case. Although the coteachers seldom had formal and regular meetings to discuss the program before each lesson, their communication was excellent. It did not appear to be an obstacle to influence their instruction and collaboration because the teachers enjoyed one another's company.

### Effective Use of Continuous Assessment

From the interviews, the team believed that good and comprehensive assessment was not just about grading and examinations. Although there was a written examination for students, the students were examined on the application, but not memorization, of the concepts derived from the Project P.A.T.H.S. In addition, continuous assessment was exercised and it played an important part in the teaching-learning process. The team found that this kind of assessment strategy was a good method to improve the students' learning and classroom participation.

As far as the implications of the present findings for future implementation of positive youth development programs are concerned, several conclusions can be highlighted. First, administrative support, including support from principals, revisions of school policy, and curriculum are important to the successful implementation of a school-based program. However, although the findings showed that irregular informal meetings were sufficient in the present case, the need for regular formal meetings should be reconsidered. Second, the involvement of dedicated teachers who share the philosophy of the program and are passionate about young people is indispensable for successful program implementation. Third, the positive effects of self-disclosure by the program implementers were illustrated in the present case study. Fourth, coteaching can facilitate success in program implementation. Finally, this case illustrates the value and viability of having continuous assessment in the implementation process.

### Limitations of the Study

Despite its rich content, there are several limitations of the present study. First, the case study was based on convenience sampling, thus limiting its generalizability to different populations. However, as Yin[35] argued, "case studies, like experiments, are generalizable to theoretical propositions and not to populations or universes" (p. 10). Second, as face-to-face interviews were adopted for the case study, demand characteristics might be present. However, as the interviews were conducted by colleagues with social work and counseling backgrounds, the informants were encouraged to voice their views (including those that were negative in nature), which could partly dismiss this criticism. Third, as the data were coded and analyzed by the second and third authors, researchers' biases might influence the interpretation of data. Nevertheless, as the authors who were involved in data analyses maintained a disciplined stand (e.g., conscious awareness and reflection of their personal biases) and the first author was not involved in the data analyses, the influence of bias on the interpretations may not be strong. Finally, as only the school coordinator and four teachers were involved, the views collected might not reflect the whole picture of the school's situation. The picture would become more complete if the school principal, senior administrators, and other teachers in the school had participated in the interviews.

### ACKNOWLEDGMENTS

The preparation for this paper and Project P.A.T.H.S. were financially supported by The Hong Kong Jockey Club Charities Trust.
